# Identifying Coevolving Partners from Paralogous Gene Families

**DOI:** 10.4137/ebo.s621

**Published:** 2008-04-24

**Authors:** Chen-Hsiang Yeang

**Affiliations:** Simons Center for Systems Biology, Institute for Advanced Study, Princeton, NJ 08540, U.S.A

## Abstract

Many methods have been developed to detect coevolution from aligned sequences. However, all the existing methods require a one-to-one mapping of candidate coevolving partners (nucleotides, amino acids) a priori. When two families of sequences have distinct duplication and loss histories, finding the one-to-one mapping of coevolving partners can be computationally involved. We propose an algorithm to identify the coevolving partners from two families of sequences with distinct phylogenetic trees. The algorithm maps each gene tree to a reference species tree, and builds a joint state of sequence composition and assignments of coevolving partners for each species tree node. By applying dynamic programming on the joint states, the optimal assignments can be identified. Time complexity is quadratic to the size of the species tree, and space complexity is exponential to the maximum number of gene tree nodes mapped to the same species tree node. Analysis on both simulated data and Pfam protein domain sequences demonstrates that the paralog coevolution algorithm picks up the coevolving partners with 60% 88% accuracy. This algorithm extends phylogeny-based coevolutionary models and make them applicable to a wide range of problems such as predicting protein-protein, protein-DNA and DNA-RNA interactions of two distinct families of sequences.

## Introduction

Coevolution of molecular components has been widely used to study the structures and functions of bio-molecules. Selective constraints operate on the entire molecular system, which often require coordinated changes of its components. These coordinated changes are manifested on the covariation of their DNA, RNA or protein sequences. In functional RNAs (e.g. ribosomal and transfer RNAs), interacting nucleic acid pairs in the secondary structure undergo compensatory changes between Watson-Crick (AU and CG) and GU base pairs (Noller and Woese, 1981; Gutell, Noller and Woese, 1986; Rzhetsky, 1995; Knudsen and Hein, 1999; Eddy, 2001; Pedersen et al. 2006). Furthermore, coordinated changes between non-standard Watson-Crick and GU pairs are found in the tertiary interactions (Noller, 2005; Dutheil et al. 2005; Yeang et al. 2007). In proteins, previous studies indicate coevolving sites within or between proteins are physically interacting (Pollock, Taylor and Goldman, 1999; Atchley et al. 2000; Tillier and Liu, 2003), energetically coupled (Lockless and Ranganathan, 1999), and located at the functionally important sites (Fares and Travers, 2006; Yeang and Haussler, 2007).

A large number of methods have been proposed to detect coevolving components from multiple sequence alignments. These methods fall into two general categories. Non-parametric methods calculate various covariation metrics of aligned sequences, including mutual information (Atchley et al. 2000; Tillier and Liu, 2003; Ramani and Marcotte, 2003; Gloor et al. 2005), correlation coeffcients (Goh et al. 2000; Dutheil et al. 2005; Fares and Travers, 2006), and the deviance between marginal and conditional distributions (Lockless and Ranganathan, 1999). Alternatively many authors have explicitly adopted parametric models of continuous-time Markov processes (CTMP) for sequence substitution and applied a hypothesis testing framework to determine coevolution (e.g. Pagel, 1994; Rzhetsky, 1995; Pollock, Taylor and Goldman, 1999; Knudsen and Hein, 1999; Barker and Pagel, 2005; Pedersen et al. 2006; Yeang et al. 2007). Given the CTMP parameters, aligned sequences and their phylogenetic tree, we can calculate the likelihood ratio between the coevolutionary and null models and use it to predict the coevolving sites.

Both parametric and non-parametric methods require a one-to-one mapping of candidate coevolving partners a priori. This is straightforward for intra-molecular interactions when there is only one family of sequences. Problems arise for inter-molecular interactions when two families of homologous molecules (RNAs, genes, protein domains) have distinct phylogenetic trees. Due to different gene duplication and loss histories, one molecule may have multiple choices of coevolving partners (when the coevolving partner in the ancestral species undergoes one or multiple duplications in the contemporary species) or no possible coevolving partner at all (when the coevolving partner in the ancestral species is lost in the contemporary species). Even when there is one gene from each family in a contemporary species, they are not necessarily coevolved if they belong to the noninteracting paralogous lineages. Identification of the coevolving partners from each species and the coevolving joint tree from the individual gene trees is computationally non-trivial and has rarely been explored by previous methods of coevolutionary analysis. Most previous works avoid this problem by restricting to intra-molecular coevolution (e.g. Tillier and Liu, 2003; Fares and Travers, 2006), manually picking the members (e.g. Goh et al. 2000; Gloor et al. 2005), or adopting simple heuristics to choose the paralogous lineages that maximize the coverage in the species tree (e.g. Yeang and Haussler, 2007). Exhaustive search on all possible selections, albeit accurate, is intractable as the number of possible combinations is exponential to the number of species.

In this work we propose an algorithm to simultaneously identify the coevolving partners and compute the likelihood score from two families of sequences that have distinct phylogenetic trees. We first apply reconciliation to map each gene tree (the phylogenetic tree of each family of sequences) to a reference species tree (the phylogenetic tree of the species where they reside). For each species tree node, we de fine a joint state of the sequence composition of gene tree nodes in each family and the assignment of the coevolving partners. A continuous-time Markov model for the sequence substitution of the two families is constructed. The coevolving subtree follows a joint coevolutionary CTMP model, whereas the remaining members of the two families are independently evolved. The choice of the coevolving subtree is dictated by the assignment of coevolving partners in each node. We then recursively apply dynamic programming to maximize the likelihood over the selection of coevolving partners and sum over possible sequence composition. The algorithm guarantees to find a maximum likelihood coevolving subtree. Time complexity is polynomial in the size of the species tree and space complexity is exponential in the maximum number of gene tree nodes mapped to the same species tree node. Our algorithm successfully picks up the coevolving partners on simulated data with 60% 88% accuracy. Moreover, on Pfam protein domain sequences our algorithm accurately identifies the domain pairs belonging to the same proteins.

The algorithm extends the power of existing CTMP models to two families of sequences with distinct phylogenetic trees. This extension is essential for detecting any inter-molecular interactions since almost all gene (protein, protein domain, functional RNA) families undergo different duplication and deletion histories. It can be applied to a wide range of problems, such as predicting protein-protein, protein-DNA and DNA-RNA interactions.

## Methods

### Overview of the algorithm

The paralog coevolution algorithm is based on the following hypotheses. First, both the species tree and the gene trees of the two families are correct. Second, the mapping from each node of a gene tree to the species tree – reconciliation – is correct. Third, in each ancestral or contemporary species, there is at most one pair of coevolving partners. Fourth, coevolving partners are the gene tree children of the coevolving partners in their parent species, unless they are the roots. Fifth, the sequence substitution of the coevolving subtree follows a joint CTMP of a given coevolutionary model, whereas the sequence substitution of the remaining parts of each gene tree follows an independent CTMP. Hypothesis 1 is the the premises of all phylogenetic tree-based models. Hypothesis 2 allows us to apply a reconciliation algorithm to find the mapping from gene trees to the species tree. Hypotheses 3 and 4 simplify the problem and make it amenable for dynamic programming. Hypothesis 5 is the premises of all CTMP models for coevolution.

The inputs of the algorithm include the species tree and the gene trees of both families, the sequences on the leaves of each gene tree, and the substitution rate matrices for single components and the pairwise coevolutionary model. Given these inputs and an assignment of coevolving partners in each species node, the likelihood of the data is the product of the likelihoods on the coevolving subtree and the independent parts. The objective is to find the coevolving partner assignment that maximizes the joint likelihood score. The outputs of the algorithm are the coevolving partner assignment at each species node and the log likelihood ratio between the coevolutionary and the null models.

We construct an augmented CTMP model on the species tree. A joint state (*S_N_*__1__(*A*),π*_N_*__1__(*A*), *S_N_*__1__ (*B*), π*_N_*__1__ (*B*)) of a species node *N*_1_ constitutes the sequence composition *S_N_*__1__ (*A*) and *S_N_*__1__ (*B*) of the gene tree nodes from families *A* and *B* mapped to N_1_, and the assignment (π*_N_*__1__ (*A*), π*_N_* __1__ (*B*)) of a pair of coevolving partners from these gene tree nodes. The evolution of the joint states follows hypotheses 4 and 5. Suppose *N*_2_ is a child node of *N*_1_ in the species tree and (*S* *_N_*__2__ (*A*), π *_N_*__2__ (*A*), *S* *_N_*__2__ (*B*), π*_N_*__2__ (*B*)) its joint state. According to hypothesis 4, the partner assignment (π*_N_*__2__ (*A*), π*_N_*__2__ (*B*)) on node *N*_2_ is compatible with (π*_N_*__2__ (*A*), π*_N_*__2__ (*B*)) only if π*_N_*__2__ (*A*) and π *_N_*__2__ (*B*) are the children of π *_N_*__1__ (*A*) and π*_N_*__1__ (*B*) in the gene trees of and *B* respectively. According to hypothesis 5, the sequence substitution from the coevolving partners (π*_N_*__1__ (*A*), π*_N_*__1__ (*B*)) on *N*_1_ to the coevolving partners (π*_N_*__2__ (*A*), π*_N_*__2__ (*B*)) on *N*_2_ follows the joint CTMP model, whereas the sequence substitution of the remaining nodes follows the independent CTMP model.

Similar to the coevolutionary CTMP model on a single phylogenetic tree, the likelihood of the observed sequences conditioned on the joint state of a species node can be recursively expressed by the conditional likelihoods of its children (equation 9). It maximizes over the assignments of coevolving partners and sums over the possible sequence states of the internal nodes. A variation of the standard dynamic programming algorithm (Felsenstein, 1981) can effciently evaluate these conditional probabilities. Furthermore, by iteratively fixing the assignment on each species node and applying dynamic programming conditioned on the fixed assignments, we can identify a set of optimal assignments. This is similar to finding a MAP configuration of loopless factor graph models using max-product (Kschischang et al. 2001).

Similar to other CTMP models, the input sequences of our algorithm are the sequences for a pair of positions in the two families. In simulation analysis, we generated the sequences of 10 position pairs from the coevolutionary model, applied the paralog coevolution algorithm to the sequences of all position pairs and combined these results to determine coevolving partners. In the analysis of real data, we treated each position pair independently, since the number of candidate position pairs for each domain family pair varies substantially and the prediction results may be affected by the number of position pairs.

### Continuous-time markov models

The sequence composition of a single nucleic or amino acid is modeled by a continuous-time Markov process (Yang, 1995). Denote by *x*(*t*) the sequence composition at time *t*. **P**(*x* (*t*)) is a probability vector of *x*(*t*) and follows a Markov process at an infinitesimal time interval:

(1) $$\frac{dP(x(t))}{dt}=P(x(t))Q.$$

where **Q** is a substitution rate matrix. Each row of **Q** must sum to 0 in order to make components of **P**(*x*(*t*)) sum to 1. We use the HKY model of nucleotide substitution (Hasegawa, Kishino and Yano, 1985) and the Dayhoff matrix of amino acid substitution (Dayoff et al. 1978). The transition probability **P**(*x*(*t*)| *x*(0)) at a finite time interval *t* is given by the matrix exponential *e***^Q^***^t^*, which is the solution of equation 1:

(2) $$P\left(x(t)=b|x(0)=a\right)=e^{Qt}[a,b].$$

Define **x**(*t*) =(*x*_1_(*t*), *x*_2_(*t*)) as the joint state of two components. The joint rate matrix **Q**_2_ is much bigger (16 × 16 for nucleotide pairs and 400 × 400 for amino acid pairs). If two sites are independently evolved, then the joint rate matrix **Q**_2_ can be derived from the rate matrix of single sites (Pagel, 1994):

(3) $$\begin{array}{l} Q_{2}^{i}\left[\left(a_{1},a_{2}),(b_{1},b_{2}\right)\right]\\ =\{\begin{array}{ll} Q[a_{1},b_{1}] & \text{if }a_{2}=b_{2},\\ Q[a_{2},b_{2}] & \text{if }a_{1}=b_{1},\\ -Q[a_{1},b_{1}]-Q[a_{2},b_{2}] & \text{if }a_{1}=b_{1},\;a_{2}=b_{2},\\ 0 & \text{otherwise}. \end{array} \end{array}$$

**Q**_2_*^i^*[(*a*_1_, *a*_2_), (*b*_1,_ *b*_2_)] specifies the sequence substitution rate of the independent model from state (*a*_1_, *a*_2_) to state (*b*_1_, *b*_2_). In **Q**_2_*^i^*, the rate of a single site change is equal to the corresponding rate in the single site rate matrix **Q**, and the rates of double site changes are all zero.

The joint rate matrix of two coupled sites has much fewer constraints, thus is difficult to estimate and subject to over-fit the limited data. To capture the covariational nature of coevolution we adopt a simple reweighting scheme on the joint rate matrix of independent evolution. It penalizes single transitions and rewards double transitions in the rate matrix:

(4) $$Q_{2}^{c}\left[\left(a_{1},a_{2}),(b_{1},b_{2}\right)\right]=\{\begin{array}{ll} ɛQ_{2}^{i}[(a_{1},a_{2}),(b_{1},b_{2})] & \text{if }a_{1}=b_{1}\;\text{or }a_{2}=b_{2},\\ r_{\left(a_{1},a_{2}\right)} & \text{if }a_{1}\neq b_{1}\;\text{and }a_{2}\neq b_{2},\\ -\sum_{\left(b_{1}^{\prime },b_{2}^{\prime })\neq (a_{1},a_{2}\right)}Q_{2}^{c}[(a_{1},a_{2}),(b_{1}^{\prime },b_{2}^{\prime })] & \text{if }a_{1}=b_{1},a_{2}=b_{2}. \end{array}$$

where ε is the penalty for single transitions and *r*_(_*_a_*__1_,_ *_a_*__2_)_ the reward for double transitions. *r*_(_*_a_*__1_,_ *_a_*__2_)_is equal for all the double transitions from the same state (*a*_1_, *a*_2_) and forces the diagonal entries in **Q**_2_*^c^* to be identical to the independent model **Q**_2_*^c^*. This simple reweighting scheme applies to both nucleotide and protein sequences, requires no assumption about the coevolving states, and has only one extra free parameter (ε). Previously we successfully detected RNA secondary and tertiary interactions (Yeang et al. 2007) and protein structural/functional constraints (Yeang and Haussler, 2007) using this model. Nevertheless, we shall emphasize the choice of the CTMP substitution rate matrix is independent of the assignment optimization algorithm. The assignment optimization algorithm can take any consistent substitution rate matrix such as the ones used in Pagel, 1994; Rzhetsky, 1995; Pollock, Taylor and Goldman, 1999; and Knudsen and Hein, 1999.

### Reconciliation and simplification of gene-species mapping

The phylogenetic trees of a family of homologous genes and the species they reside are often different due to the duplication and loss of genes. Reconciliation maps each node in the gene tree to a node in the species tree, and designates it as a duplication or speciation event. We adopt a variation of the parsimonious reconciliation algorithm by Zmasek and Eddy, 2001. Briefly, it recursively maps nodes in the gene tree to nodes in the species tree. An internal gene tree node *g* is mapped to species tree node ℳ(*g*), the most recent common ancestor of species tree nodes ℳ (*g*_1_) and ℳ (*g*_2_) mapped from *g*’s children *g*_1_ and *g*_2_· *g* is a duplication node if ℳ (*g*) = ℳ (*g*_1_) or ℳ (*g*) = ℳ (*g*_2_). Figure 1.1 illustrates reconciliation. The quality of the parsimonious reconciliation has been challenged and various alternative approaches have been proposed (e.g. Arvestad et al. 2003, Berglund-Sonnhammer et al. 2006). We chose the parsimonious reconciliation for its simplcity, and this choice is again independent of the assignment optimization algorithm. Other reconciliation methods can be applied to generate the mapping from gene trees to the species tree.

A pre-requisite of our algorithm is that sequence evolution of nodes in a gene tree can be expressed as the joint state transitions of nodes in the species tree. When multiple nodes along a path of a gene tree are mapped to the same species node, the internal transitions of those nodes cannot be captured by the joint state transitions in the species tree (Fig. 1.2). Complex states of partner assignments are needed if hierarchies of gene tree nodes are mapped to the same species tree node. Pairs of nodes, paths, or nodes and paths can be selected as coevolving partners. To avoid this complexity we have to flatten the hierarchies mapped to the same species node into one layer and update the branch length accordingly(Fig. 1.3). This is done by the following procedures. From bottom of the species tree, identify the “forest” of gene tree nodes mapped to the same species node, and find the top layer of these gene tree nodes. Collapse all the nodes below the top layer, such that the top layer gene tree nodes mapped to one species node directly link to the top layer gene tree nodes mapped to its child species node. The branch length between two top layer gene tree nodes is the path length between them in the uncollapsed tree.

### Evaluating marginal likelihoods

We first define the following notations. Let *A* and *B* be the two families of genes, *T_A_*, *T_B_* their gene trees, and *T* the species tree. Denote *pa*(*g*) and *ch*(*g*) as the parent and children of *g*. For each species tree node *N* ∈ *T*, let *N*(*A*) ={*g* ∈ *T_A_* : ℳ (*g*) = *N*} and *N*(*B*) = {g ∈ *T_B_* : ℳ (*g*) = *N*}be the gene tree nodes mapped to *N*. Let 𝒞 be the alphabet set (nucleotides or amino acids). For each species tree node *N*, denote *S_N_* (*A*) ∈ 𝒞^|^*^N^* ^(^*^A^*^)|^ and *S_N_* (*B*) ∈ 𝒞^|^*^N^* ^(^*^B^*^)|^ as the sequence composition of *N*(*A*) and *N*(*B*). In addition, let π*_N_* (*A*) ∈*N* (*A*) ∪ φ and π*_N_* (*B*) ∈*N* (*B*) ∪ φ be the assignment of coevolving partners of *A* and *B* in species tree node *N*. Define *J_N_* = (*S_N_* (*A*), π*_N_* (*A*), *S_N_* (*B*), π*_N_* (*B*) the joint state of sequences and assignments of the gene tree nodes mapped to *N*. Finally, denote *D_N_* as the sequence composition of all the *A* and *B* genes mapped to the descendant leaves of *N*, and define *D_g_* of a gene tree node *g* as the sequence composition of *g*’s descendants leaves in the gene tree.

**Figure 2:**
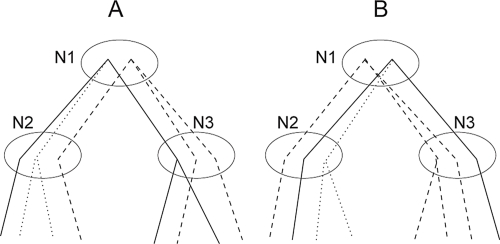
Evaluating the conditional likelihood. Solid: the coevolving portion. Dashed: the independent portion starting at *N*__1__. Dotted: the independent portion of the siblings of the coevolving nodes at *N*__2__ and *N*__3__

Consider the subtrees in Figure 2. The conditional probability *P*(*D_N_*__1__ |*J_N_*__1__) specifies the likelihood of observing the gene sequences in the descendants of *N*_1_ conditioned on the joint state of *N*_1_. It is calculated by summing over all possible sequences and maximizing over all possible assignments of the internal nodes connecting *N*_1_ and its descendants.

The conditional likelihood can be expressed by a recursive formula:

(5) $$\begin{array}{l} P(D_{N_{1}}|J_{N_{1}})=\max_{\left(\pi_{N_{2}}(A),\pi_{N_{2}}(B))≺(\pi_{N_{1}}(A),\pi_{N_{1}}(B)\right)}\sum_{S_{N_{2}}(A),S_{N_{2}}(B)}P(J_{N_{2}}|J_{N_{1}})P(D_{N_{2}}|J_{N_{2}})\cdot \\ \max_{\left(\pi_{N_{3}}(A),\pi_{N_{3}}(B))≺(\pi_{N_{1}}(A),\pi_{N_{1}}(B)\right)}\sum_{S_{N_{3}}(A),S_{N_{3}}(B)}P(J_{N_{3}}|J_{N_{1}})P(D_{N_{3}}|J_{N_{3}}) \end{array}$$

where ≺ denotes that the assignment in a child species node is compatible with the assignment in its parent. According to hypothesis 4, (π*_N_*__2__ (*A*), π*_N_*__2__ (*B*)) ≺ (π*_N_*__1__ (*A*), π*_N_*__1__ (*B*)) if *pa*(π*_N_*__2__ (*A*)) = π*_N_*__1__ (*A*) and *pa*(π*_N_*__2__ (*B*)) = π*_N_*__1__ (*B*), or that π*_N_*__2__ (*A*) = φ and π*_N_*__2__ (*B*) = φ.

Conditional probability *P*(*J_N_*__2__|*J_N_*__2__) can be factorized as

(6) $$\begin{array}{l} P(J_{N_{2}}|J_{N_{1}})\\ =P(\pi_{N_{2}}(A),\pi_{N_{2}}(B)|\pi_{N_{1}}(A),\pi_{N_{1}}(B))\\ P(S_{N_{2}}(A),S_{N_{2}}(B)|J_{N_{1}},\pi_{N_{2}}(A),\pi_{N2}(B)). \end{array}$$

where the conditional probability *P*(π*_N_*__2__ (*A*), π*_N_*__2__ (*B*)| π*_N_*__1__ (*A*), π*_N_*__1__ (*B*)) is uniform over all the compatible assignments (π*_N_*__2__ (*A*), π*_N_*__2__ (*B*)) ≺ (π*_N_*__1__ (*A*), π*_N_*__1__ (*B*)) and 0 for incompatible assignments.

Given the assignments of the parent (*N*_1_) and one child (*N*_2_), the *N*_2_ part in equation 5 is the product of three terms (Fig. 2):

1. The coevolving portion from (π*_N_*__1__ (*A*), π*_N_*__1__ (*B*)) to the descendants of (π*_N_*__2__ (*A*), π*_N_*__2__ (*B*)).
2. The independent portion of the nodes in *N*(*A*)\ π*_N_*__1__ (*A*) and *N*(*B*)\ π*_N_* __1__ (*B*).
3. The independent portion of the siblings of π*_N_*__2__ (*A*) and π*_N_*__2__ (*B*) in *N*_2_.

Notice the second term is common for both *N*_2_ and *N*_3_ since it is independent of the assignment in *N*_2_ and *N*_3_. It has to be evaluated only once. If π*_N_*__1__ (*A*) = φ and π*_N_*__1__ (*B*) = φ, then only the second term is valid. If π*_N_*__1__ (*A*) ≠ φ, π*_N_*__1__ (*B*) ≠ φ π*_N_*__2__ (*A*) = φ and π*_N_*__2__ (*B*) = φ, then the second and the third terms are valid.

The first term follows the recursive formula of the coevolutionary model:

(7) $$\begin{array}{l} \sum_{S_{\pi_{N_{2}(A)}}(A),S_{\pi_{N_{2}(B)}}(B)}\cdot \\ P(S_{\pi_{N_{2}(A)}}(A),S_{\pi_{N_{2}(B)}}(B)|S_{\pi_{N_{1}(A)}}(A),S_{\pi_{N_{1}(B)}}(B))\cdot \\ P(D_{\pi_{N_{2}(A)}(A),}{}_{\pi_{N_{2}(B)}(B)}|S_{\pi_{N_{2}(A)}}(A),S_{\pi_{N_{2}(B)}}(B)). \end{array}$$

Conditional probability *P*(*S*_π *_N_*_2__(_*_A_*_)__ (*A*),*S*_π*_N_*_2__(_*_B_*_)__(*B*)|*S*_π*_N_*_1_ (_*_A_*_)_ (*A*), *S*_π*_N_*_1__(_*_B_*_)__ (*B*)) is calculated using equations 2 and 4. *P*(*D*_π*_N_*_2__(_*_A_*_)_ (_*_A_*_)_, _π*_N_*_2__(_*_B_*_)_(_*_B_*_)_| *S*_π*_N_*_2__(_*_B_*_)__(*B*)) is the coevolving portion of *P*(*D_N_*__2__|*J_N_*__2__). It can be obtained by dividing *P*(*D_N_*__2__|*J_N_*__2__) by the independent portion of *P*(*D_N_*__2__|*J_N_*__2__). Thus

(8) $$\begin{array}{l} P(D_{\pi_{N_{2}(A)}(A),\pi_{N_{2}(B)}(B)}|S_{\pi_{N_{2}(A)}}(A),S_{\pi_{N_{2}(B)}}(B))\\ =\frac{P(D_{N_{2}}|J_{N_{2}})}{\prod_{i\in N_{2}(A)\backslash \pi_{N_{2}(A)}}P(D_{i}|S_{i})\prod_{j\in N_{2}(B)\backslash \pi_{N_{2}(B)}}P(D_{i}|S_{i})}. \end{array}$$

for any joint state *J_N_*__2__where *P*(*D_N_*__2__|*J_N_*__2__) > 0. *P*(*D_N_*__2__|*J_N_*__2__) is already computed, and the terms in the denominator can be evaluated by standard dynamic programming of single components.

The second and third terms in the *N*_2_ part of equation 5 are the product of the likelihoods of single components and can be effciently calculated. The evaluation of the *N*_3_ part of equation 5 follows the same procedure. By combining these terms, equation 5 is reduced to

(9) $$\begin{array}{l} P(D_{N_{1}}|J_{N_{1}})\\ =\prod_{i\in N_{1}(A)\backslash \pi_{N_{1}(A)}}P(D_{i}|S_{i})\cdot \prod_{i\in N_{1}(B)\backslash \pi_{N_{1}(B)}}P(D_{j}|S_{j})\cdot \\ \max_{\left(\pi_{N_{2}}(A),\pi_{N_{2}}(B))≺(\pi_{N_{1}}(A),\pi_{N_{1}}(B)\right)}\\ P(\pi_{N_{2}}(A),\pi_{N_{2}}(B)|\pi_{N_{1}}(A),\pi_{N_{1}}(B))\cdot \\ \sum_{S_{\pi_{N_{2}(A)}}(A),S_{\pi_{N_{2}(B)}}(B)}\cdot \\ P(S_{\pi_{N_{2}(A)}}(A),S_{\pi_{N_{2}(B)}}(B)|S_{\pi_{N_{1}(A)}}(A),S_{\pi_{N_{1}(B)}}(B))\cdot \\ \frac{P(D_{N_{2}}|J_{N_{2}})}{\prod_{i\in N_{2}(A)\backslash \pi_{N_{2}(A)}}P(D_{i}|S_{i})\cdot \prod_{j\in N_{2}(B)\backslash \pi_{N_{2}(B)}}P(D_{j}|S_{j})}\cdot \\ \prod_{i\in ch\left(\pi_{N_{1}(A)}\right)\backslash \pi_{N_{2}}(A)}P(D_{i}|S_{i})\cdot \\ \prod_{j\in ch\left(\pi_{N_{1}(B)}\right)\backslash \pi_{N_{2}}(A)}P(D_{j}|S_{j})\cdot \\ \max_{\left(\pi_{N_{3}}(A),\pi_{N_{3}}(B))≺(\pi_{N_{1}}(A),\pi_{N_{1}}(B)\right)}\cdot \\ P(\pi_{N_{3}}(A),\pi_{N_{3}}(B))|\pi_{N_{1}}(A),\pi_{N_{1}}(B))\cdot \\ \sum_{S_{\pi_{N_{3}(A)}}(A),S_{\pi_{N_{3}(B)}}(B)}\cdot \\ P(S_{\pi_{N_{3}(A)}}(A),S_{\pi_{N_{3}(B)}}(B)|S_{\pi_{N_{1}(A)}}(A),S_{\pi_{N_{1}(B)}}(B))\cdot \\ \frac{P(D_{N_{3}}|J_{N_{3}})}{\prod_{i\in N_{3}(A)\backslash \pi_{N_{3}(A)}}P(D_{i}|S_{i})\cdot \prod_{j\in N_{3}(B)\backslash \pi_{N_{3}(B)}}P(D_{j}|S_{j})}\cdot \\ \prod_{i\in ch(\pi_{N_{1}(A)})\backslash \pi_{N_{3}}(A)}P(D_{i}|S_{i})\cdot \\ \prod_{j\in ch(\pi_{N_{1}(B)})\backslash \pi N_{3}(A)}P(D_{j}|S_{j}). \end{array}$$

By applying equation 9 recursively we can calculate the conditional likelihood *P*(*D_N_* |*J_N_* ) for each species tree node.

### Finding the optimal assignments

An optimal assignment (π*_N_*__1__ (*A*), π*_N_*__1__ (*B*), …,π*_N_*_*_n_*_(*A*), π*_N_*_*_n_*_ (B)) maximizes the marginal likelihood *P*(*D*|π*_N_*__1__ (*A*), π*_N_*__1__ (*B*), …, π*_N_*_*_n_*_ (*A*), *N_n_* (*B*)) of the observed sequences. The maximum likelihood is equal to

(10) $$\begin{array}{l} \underset{\pi_{N_{r}}(A),\pi_{N_{r}}(B)}{\max}\cdot \\ \sum_{S_{N_{r}}(A),S_{N_{r}}(B)}P(S_{N_{r}}(A),S_{N_{r}}(B))P(D_{N_{r}}|J_{N_{r}}). \end{array}$$

where *N_r_* is the root node of *T* and *P*(*S_N_*_*_r_*_) the prior probability of sequence composition for gene tree nodes mapped to *N_r_*. Thus the optimal assignment (π̂*_N_*_*_r_*·_ (*A*), π̂*_N_*_*_r_*_ (*B*)) at the root can be obtained by maximizing equation 10.

To find the optimal assignments of other nodes, we iteratively fix the assignments of ancestral nodes and calculate equation 10 conditioned on the fixed assignments. This leads to the following algorithm:

1. Set current node *N* = *N_r_*, the root node of *T*, evidence *E* = φ.
2. Calculate the function ψ (π*_N_*(*A*), π *_N_*(*B*)|*E* ) =∑*_S_*_*_N__r_*(_*_A_*_), S*_N__r_*(B)_ *P*(*S_N_*_*_r_*_ (*A*), *S_N_*_*_r_*_ (*B*))*P*(*D_N_*_*_r_*_|*J_N_*_*_r_*_, *E*) conditioned on *E* and each possible assignment of π*_N_*(*A*), π*_N_*(*B*). Find (π̂*_N_*(*A*), π̂*_N_*(*B*)) = arg max_π*_N_*(_*_A_*_), π *_N_*(B)_ ψ(π*_N_*(A), π*_N_* (B) | E).
3. *E* = *E* ∪{(π̂*_N_* (*A*), π̂*_N_* (*B*))}. Descend to a child *N_c_* of *N*.
4. Iteratively repeat 2–3 until all nodes are fixed. ψ(π*_N_*(*A*), π*_N_*(*B*) | *E)* uses the recursive equation 9 to calculate *P*(*D_N_*_*_r_*_ |*J_N_*_*_r_*_,*E*) except fixing the assignments in *E* instead of maximizing them.

#### Proposition

Assignment *E* obtained from the recursive algorithm is an optimal assignment of *P*(*D*|π*_N_*__1__ (*A*), π*_N_*__1__ (*B*), …, π*_N_*_*_r_*_ (*A*), π*_N_*_*_r_*_ (*B*)).

#### Proof Sketch

Equation 9 is exact since the model structure is a tree. Initially, *E* = φ is contained in a global optimal assignment. At each step of the iteration, it can be shown that

(11) $$\begin{array}{l} \psi \left(\pi_{N}(A),\pi_{N}(B)|E\right)\\ =\underset{\pi \backslash (E\cup N)}{\max}P\left(D|\pi_{N}(A),\pi_{N}(B),\pi \backslash (E\cup N),E\right). \end{array}$$

where π\(*E* ∪ *N*) denotes the assignments of all nodes except the fixed assignments *E* and the current node *N*. Clearly, if *E* is contained in a global optimal assignment, then the optimal (π*_N_* (*A*), π*_N_* (*B*)) obtained from ψ (π_N_(*A*), π_N_(*B*) | *E)* is also contained in a global optimal assignment. Otherwise (π*_N_*(*A*), π*_N_*(*B*)) would be replaced by the assignment of *N* in the global optimum. By induction the final *E* obtained from the recursive algorithm is a global optimal assignment. Nevertheless, multiple optimal assignments may exist and the recursive algorithm can only find one of them. Q.E.D.

### Time and space complexity

The computational bottlenecks of the algorithm are matrix exponentiation and multiplication. We apply the Padé polynomial approximation to compute matrix exponentials (Sidje, 1998), whose time complexity is cubic to the dimension of the matrix. Denote *n*, *n_A_* and *n_B_* as the number of nodes in the species and gene trees, and *k* the maximum number of gene tree nodes mapped to the same species tree node. Passing messages of the entire tree (equation 9) requires *n* exponentiation of the joint rate matrix (dimension |𝒞|^2^ × |𝒞|^2^), *n_A_* + *n_B_* exponentiation of the single rate matrix (dimension |𝒞| ×|𝒞|), |𝒞|^4^ · *n* multiplications on the coevolving part and |𝒞|^2^ · (*n_A_* + *n_B_*) multiplications on the independent part. *n* complete message passings are required to fix the assignment in each node. Hence time complexity is *O*(|𝒞|^6^ *n*^2^). Time complexities of reconciliation and gene tree flattening are *O*(*n_A_* + *n*_B_) and *O*(*nk*) respectively and are negligible.

Albeit quadratic in the size of the species tree, the computational time is long for protein sequences (|𝒞| = 20). We alleviate the problem by quantizing branch lengths into a small number of intervals and computing the matrix exponentiation on quantized branch lengths. The conditional probability matrices on quantized branch lengths are precomputed, stored and used repetitively along each branch in each iteration. This simplification reduces the time cost of exponentiation to a constant (*O* (|𝒞|^6^ · *q*), *q* is the number of quantized intervals), and the overall time complexity becomes *O*(|𝒞|^4^ · *n*^2^). However, the likelihood score is no longer accurate since the conditional probabilities are approximations.

The algorithm stores *P* (*D_N_* | *J_N_*) for each joint state on each species tree node. There are at most |𝒞|^2^*^k^* sequence states and *k*^2^ assignment states for each node. Space complexity is the number of joint states of the species tree and is *O*(*nk*^2^|𝒞|^2^*^k^*). The space complexity for the pre-computed matrix exponentials on quantized branch lengths is *O*(|𝒞|^4^ · *q*) and negligible.

## Results

As a proof-of-concept demonstration we applied the paralog coevolution algorithm first to a simulated dataset and then to aligned protein domain sequences from the Pfam database (Bateman et al. 2002). On simulated data the paralog coevolution algorithm identified the coevolving partners with 73%–88% accuracy. More strikingly, on aligned protein domain sequences the algorithm identified the domain pairs belonging to the same proteins with a similar range of accuracy rate. The paralog coevolution algorithm significantly outperforms random assignments on both simulated and real data.

### Analysis on simulation data

We first applied the paralog coevolution algorithm on simulated data. A binary species tree and two compatible gene trees were generated by branching processes. The length between two consecutive branching events followed an exponential distribution with rate 0.01. The root of a gene tree was mapped to the root of the species tree. Each branching event in a gene tree was randomly determined as either speciation or duplication. For speciation the two children of a gene tree node were mapped to the two children of its species tree node. For duplication the children were mapped to the same species tree node. The probability ratio of duplication versus speciation events was set such that the number of genes from each family was about twice as the number of species. To reduce computational time we used RNA nucleotide alphabets (AUCG). The coevolving subtrees of the two families were chosen a priori. The sequences of the independent portion in each family were generated by a single CTMP with the HKY model. The joint sequences of the coevolving portion of the two families were generated by a coevolving CTMP process (equation 4) with ε= 0.1. In each trial we generated the sequences of 10 position pairs independently. The assignments of coevolving partners in each species were determined by the majority votes of the assignments inferred from the sequences of the 10 position pairs. Since the co-species genes with identical sequences are not distinguishable, we compared the number of mismatched sequences (instead of genes) between the reference and inferred coevolving pairs. As a comparison we randomly selected a pair of genes from each species. 100 simulated data were generated for 5, 10, 20 and 40 species respectively.

**Figure 3:**
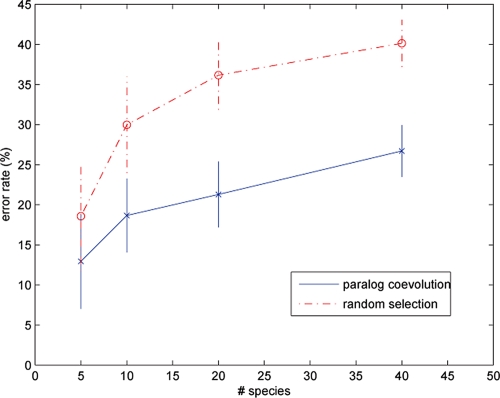
Misassignment rates on simumulated data. Vertical lines indicate 0.5 standard deviations from the mean error rates.

Figure 3 shows the mean error rates (the fraction of mismatched coevolving sequences) of the paralog coevolution algorithm and random assignments versus the number of species. Clearly, the paralog coevolution algorithm consistently outperforms random assignments when the tree size varies from 5 to 40. The error rates grow from 12% to 27% as the number of species increases from 5 to 40. This is sensible since the coevolving pairs are confounded by more false positives when the tree size increases. The error rate gap between the paralog coevolution algorithm and random assignments also increases with the number of species: 6% for 5 species and 13% for 40 species. The error rate difference is greater than one standard deviation as the number of species ≥10, suggesting the difference is statistically significant.

### Analysis on Pfam protein domain sequences

We then applied the paralog coevolution algorithm to pairs of aligned domain family sequences from the Pfam database (Bateman et al. 2002). There are 8183 domain families, 3722468 domain family pairs that co-appear in more than 20 species, and more than 1.171 × 10^11^ inter-domain position pairs. Previously we applied a large-scale screening on those 0.1 trillion position pairs and identified 3953 candidate coevolving position pairs from 582 domain family pairs (Yeang and Haussler, 2007). These position pairs passed various filterings of sequence covariation and had high scores according to the coevolutionary model (using a heuristic instead of the paralog coevolution algorithm to extract coevolving partners). Furthermore, the selected position pairs in the same proteins or protein complexes exhibited spatial proximity, and many of the coevolving positions were located at functionally important sites. Hence they are strong candidates for coevolving positions. To save time we decided to focus our search on those 3953 position pairs. The CTMP parameters were set according to the values in Yeang and Haussler, 2007. The list of those 3953 candidate position pairs are reported in the Supplementary File 1.

There is no gold standard for coevolving partners in the real data. Since domains belonging to the same proteins are more likely to coevolve, we expect to identify the co-protein domains using the paralog coevolution algorithm. Three more pre-filtering procedures were applied to further trim down the data. First, we ruled out the domain family pairs which contained co-protein domains in less than half of their members. Domain family pairs of different proteins are excluded since we cannot validate the results. Second, within each species there may exist multiple paralogous members of an identical sequence. To avoid confusion we only kept one representative from this set and removed the others. Third, to relieve the computational burden we filtered out the position pairs that generated more than 1 million joint states in at least one species node. 475 position pairs were retained after these filtering criteria.

For each position pair we calculated the optimal assignments and log-odds ratio of the aligned sequences and counted the fraction of inferred coevolving partners belonging to the same protein. As a comparison we also performed 1000 random assignments and counted the co-protein rate. Figure 4.1 shows the accuracy (co-protein) rates versus the threshold on the log-odds ratios. Clearly, a higher fraction of co-protein domains are identified by the paralog coevolution algorithm with a more stringent log-odds ratio cutoff. With threshold 10.0, 80% of the inferred coevolving partners appear in the same proteins. The accuracy rate of random assignments is uncorrelated with the log-odds ratio cutoff (as expected) and is substantially lower than the paralog coevolution algorithm. Strikingly, even on the sequences of weak coevolutionary scores (large negative values) the paralog coevolution algorithm still outperforms random assignments.

We also calculated the p-value of each paralog coevolution prediction (the fraction of random assignments exceeding the accuracy rate of the paralog coevolution prediction) and plotted the fraction of the predictions with p-value ≤0.05 versus the log-odds ratio threshold in Figure 4.2. It demonstrates the improvement of the prediction significance with an increasing threshold. With threshold 10.0 over 80% predictions are statistically significant.

The price of a stringent threshold is the reduction of coverage. Figure 4.3 shows the number of predictions passing the thresholds. With threshold 10.0 only 30 position pairs were retained.

## Discussion

As genome-scale sequences of more species become available, more information about the dependent evolution of multiple loci will be unraveled. Since gene duplication and loss are prevalent in every genome, extracting the dependency from families of paralogous/orthologous genes is critical in studying sequence evolution. Current methods of detecting sequence coevolution are primarily restricted to single families of genes. We propose an algorithm to identify coevolving partners from two paralogous families. The method builds a joint state of sequence composition and the assignment of coevolving partners, and applies dynamic programming to identify the optimal assignments. Under certain hypotheses about sequence evolution this algorithm guarantees to find the maximum likelihood assignments. Time complexity is quadratic to the size of the species tree, whereas space complexity is exponential to the maximum number of gene tree nodes mapped to the same species tree node. The algorithm outperforms random assignments on both simulated RNA and real protein sequences. On Pfam protein sequences the algorithm identifies co-protein domain pairs with up to 80% accuracy.

Despite its advantages, the algorithm has two shortcomings. First, time and space complexities are large for big trees, especially for protein sequences (high |𝒞|^6^) and the species tree with many gene tree nodes mapped to the same species node (high |𝒞|^2^*^k^*). We alleviated the problem by precomputing the matrix exponentials of quantized branch lengths. Yet these approximations also reduce the accuracy of likelihood scores. Second, the algorithm is based on several strong hypotheses about Z trees and sequence evolution. In the real data these hypotheses may not hold. For instance, reconciliation may have errors, sequence substitution of single or double components may not follow the parametric models, there may exist multiple pairs of coevolving partners in each species. A robust revision of the current algorithm to reduce space/time complexity and the requirement for the strong hypotheses are called for in the future.

## Figures and Tables

**Figure 1:**
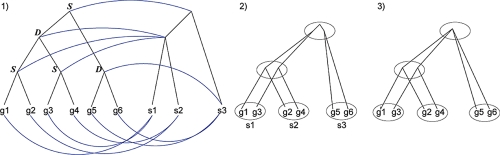
(**1**)Reconciliation of a gene tree to a species tree. S: speciation. D: duplication. (**2**) Concatenated representation of the reconciled tree. (**3**) The sub gene tree mapped to the root of the species tree is collapsed into one node.

**Figure 4:**
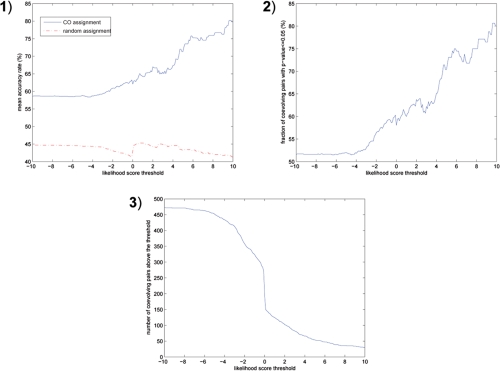
Prediction results on Pfam data, **1**) mean accuracy rate, **2**) fraction of statistically significant predictions, **3**) number of predictions above the threshold.

## References

[1] Noller HF, Woese CR (1981). Secondary structure of 16S ribosomal RNA. Science.

[2] Gutell RR, Noller HF, Woese CR (1986). Higher order structure in ribosomal RNA. EMBO J.

[3] Rzhetsky A (1995). Estimating substitution rates in ribosomal RNA genes. Genetics.

[4] Knudsen B, Hein J (1999). RNA secondary structure prediction using stochastic context-free grammars and evolutionary history. Bioinformatics.

[5] Eddy SR (2001). Non-coding RNA genes and the modern RNA world. Nat Rev Genet.

[6] Pedersen JS, Bejerano G, Siepel A, Rosenbloom K, Lindblad-Toh K, Lander ES, Kent J, Miller W, Haussler D (2006). Identification and classification of conserved RNA secondary structures in the human genome. PLoS Comp Bio.

[7] Noller HF (2005). RNA structure: reading the ribosome. Science.

[8] Dutheil J, Pupko T, Jean-Marie A, Galtier N (2005). A model-based approach for detecting coevolving positions in a molecule. Mol Biol Evol.

[9] Yeang CH, Darot JFJ, Noller HF, Haussler D (2007). Detecting the coevolution of biosequences an examples of RNA interaction prediction. Mol Biol Evol.

[10] Pollock DD, Taylor WR, Goldman N (1999). Coevolving protein residues: maximum likelihood identification and relationship to structure. J Mol Biol.

[11] Atchley WR, Wollenberg KR, Fitch WM, Terhalle W, Dress AW (2000). Correlations among amino acid sites in bHLH protein domains: an information theoretic analysis. Mol Biol Evol.

[12] Tillier ERM, Lui WH (2003). Using multiple interdependency to separate functional from phylogenetic correlations in protein alignments. Bioinformatics.

[13] Lockless SW, Ranganathan R (1999). Evolutionary conserved pathways of energetic connectivity in protein families. Science.

[14] Fares M, Travers SAA (2006). A novel method for detecting intramolecular coevolution: adding a further dimension to select constraints analyses. Genetics.

[15] Yeang CH, Haussler D (2007). Detecting coevolution in and among protein domains. PLoS Comp Biol.

[16] Ramani AK, Marcotte EM (2003). Exploiting the co-evolution of interacting proteins to discover interaction specificity. J Mol Biol.

[17] Gloor GB, Martin LC, Wahl LM, Dunn SD (2005). Mutual information in protein multiple sequence alignments reveals two classes of coevolving positions. Biochemistry.

[18] Goh CS, Bogan AA, Joachmiak M, Walther D, Cohen FE (2000). Co-evolution of proteins with their interaction partners. J Mol Biol.

[19] Pagel M (1994). Detecting correlated evolution on phylogenies: a general method for the comparative analysis of discrete characters. P Roy Entomol Soc B.

[20] Barker D, Pagel M (2005). Predicting functional gene links from phylogenetic-statistical analyses of whole genomes. PLoS Comp Biol.

[21] Felsenstein J (1981). Evolutionary trees from DNA sequences: a maximum likelihood approach. J Mol Evol.

[22] Kschischang F, Frey B, Loeliger H (2001). Factor graphs and the sum-product algorithm. IEEE trans info theory.

[23] Yang Z (1995). A space-time process model for the evolution of DNA sequences. Genetics.

[24] Hasegawa M, Kishino H, Yano T (1985). Dating the human-ape splitting by a molecular clock of mitochondrial DNA. J Mol Evol.

[25] Dayhoff MO, Schwartz RM, Orcutt BC (1978). A model of evolutionary change in proteins. Atlas of protein sequence and structure.

[26] Zmasek CM, Eddy SR (2001). A simple algorithm to infer gene duplication and speciation events on a gene tree. Bioinformatics.

[27] Arvestad L, Berglund AC, Lagergren J, Sennblad B (2003). Bayesian gene/species tree reconciliation and orthology analysis using CMC. Bioinformatics.

[28] Berglund-Sonnhammer AC, Steffansson P, Betts MJ, Liberles DA (2006). Optimal gene trees from sequences and species trees using a soft interpretation of parsimony. J Mol Evol.

[29] Sidjie RB (1998). EXPOKIT: A software package for computing matrix exponentials. ACM Trans Math Softw.

[30] Bateman A, Birney E, Cerruti L, Durbin R, Etwiller L, Eddy SR, Griffth-Jones S, Howe KL, Marshall M, Sonnhammer ELL (2002). The Pfam protein families database. Nucleic Acids Res.

